# Plasma EDA2R and Risk of Cardiovascular Diseases and All‐Cause Mortality: Analysis of the UK Biobank Cohort

**DOI:** 10.1002/clc.70314

**Published:** 2026-04-28

**Authors:** Ziqing Ruan, Jiabin Tu, Hongli Xu, Yupeng Zhi, Chun Chen, Kaiyang Lin, Yansong Guo

**Affiliations:** ^1^ Department of Cardiology Shengli Clinical Medical College of Fujian Medical University, Fujian Provincial Hospital, Fuzhou University Affiliated Provincial Hospital Fuzhou China; ^2^ Fujian Provincial Key Laboratory of Cardiovascular Disease, Fujian Provincial Center for Geriatrics, Fujian Provincial Clinical Research Center for Severe Acute Cardiovascular Diseases Fuzhou China; ^3^ Fujian Heart Failure Center Alliance Fuzhou China; ^4^ Fujian Medical University School of Pharmacy Fuzhou Fujian China

**Keywords:** all‐cause mortality, cardiovascular disease, EDA2R, GO enrichment analysis, tumor necrosis factor receptor superfamily, UK biobank

## Abstract

**Background:**

Cardiovascular disease (CVD) remains the leading cause of morbidity and mortality worldwide. Ectodysplasin A2 receptor (EDA2R), a newly identified member of the tumor necrosis factor receptor superfamily, has been implicated in metabolic and inflammatory processes, but its role in CVD is unknown.

**Objective:**

To examine the associations of plasma EDA2R levels with incident CVD and all‐cause mortality.

**Methods:**

A total of 45,305 UK Biobank participants with baseline plasma proteomics measured by Olink were included. EDA2R expression levels in the UKB have been converted to normalized protein expression (NPX). Cox proportional hazards models were used to assess the relationships between EDA2R and CVD, as well as all‐cause mortality. Temporal trajectories of EDA2R before events were examined using a nested case–control design with LOESS smoothing. Causal mediation and GO enrichment analyses were performed to identify mediating proteins and underlying pathways.

**Results:**

Over a median follow‐up of 15 years, 8667 participants (19.1%) developed CVD, and 3988 (8.8%) died. After fully adjusted, each 1 NPX increase in plasma EDA2R was associated with a 74% higher risk of CVD and a 177% higher risk of all‐cause mortality, with risks increasing monotonically across the entire distribution. Mediation analysis identified 302 proteins for CVD and 482 for mortality, enriched in death receptor activity, tumor necrosis factor receptor activity, cytokine and growth factor binding, and immune receptor activity.

**Conclusion:**

Elevated plasma EDA2R is strongly associated with long‐term risks of CVD and all‐cause mortality, suggesting its potential as a novel prognostic biomarker and therapeutic target.

## Graphical abstract

In this large‐scale UK Biobank analysis, elevated plasma EDA2R was associated with a 74% higher risk of cardiovascular disease and a 177% higher risk of all‐cause mortality, with a 7.2‐year loss in life expectancy in the highest EDA2R group, suggesting its potential as a prognostic biomarker and therapeutic target.

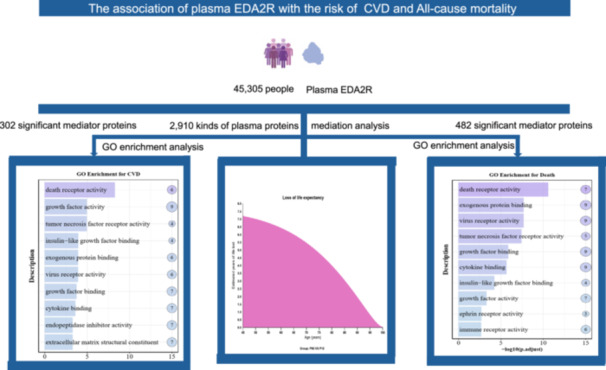

## 1 Introduction

Cardiovascular diseases (CVD) are the leading cause of death and disability worldwide, posing a serious threat to human health and imposing a substantial socioeconomic burden [1]. Although considerable progress has been made in early intervention, reperfusion therapy, and pharmacological management, the incidence of CVD remains high. Therefore, identifying novel biomarkers and potential therapeutic targets continues to be a critical focus in CVD research [2]. Therefore, there is an urgent need to discover novel biomarkers to enhance early predictive capacity, improve individualized risk stratification, and guide precise preventive and therapeutic interventions.

Ectodysplasin A (EDA) is a member of the tumor necrosis factor (TNF) superfamily. Alternative splicing of EDA produces multiple transcript variants, among which the best characterized are the isoforms EDA‐A1 and EDA‐A2. EDA‐A1 specifically binds to the EDAR receptor, whereas EDA‐A2 interacts exclusively with ectodysplasin A2 receptor (EDA2R) [3, 4]. EDA2R, also known as the X‐linked ectodysplasin receptor [5]. Initially, EDA2R was primarily associated with ectodermal development disorders such as hypohidrotic ectodermal dysplasia [6]. In recent years, EDA2R has been implicated in various metabolic diseases. It has been reported to be positively associated with an increased risk of peripheral artery disease (PAD) in patients with type 2 diabetes, and its inclusion in clinical models significantly improves PAD prediction [7]. Transcriptomic analysis (RNA‐seq) of lung tissues from 68 subjects revealed that EDA2R expression was elevated in the elderly group, suggesting a potential role as a risk factor for pulmonary aging [8]. Furthermore, an upregulation of EDA2R has been observed in tumor‐bearing mice and in patients with cancer cachexia [3]. In juvenile mice, even low‐dose cranial irradiation was sufficient to induce a marked increase in EDA2R expression [9]. Mechanistically, EDA2R has been shown to activate several canonical inflammatory signaling pathways, including NF‐κB and MAPK, thereby promoting the release of proinflammatory cytokines, regulating immune cell recruitment, and modulating the tissue microenvironment [10]. A small‐scale cohort study found that EDA2R was associated with the occurrence of CVD in HIV patients [11]. Wang et al. also discovered that EDA2R was positively correlated with the severity of ischemia‐reperfusion injury. However, there is still a lack of large‐scale cohort studies to determine the association of EDA2R with CVD and the risk of death [12].

The present study aims to elucidate the role of EDA2R in the onset and progression of CVD. Furthermore, we seek to assess its potential as a novel biomarker and therapeutic target, thereby providing a theoretical foundation for the development of future individualized prevention and treatment strategies.

## 2 Methods

### 2.1 Study Population

The UK Biobank is a large‐scale, prospective cohort study that has enrolled more than half a million adults across England, Scotland, and Wales. At baseline, participants provided information on demographics, medical history, and lifestyle through touchscreen questionnaires, nurse‐led interviews, and linkage to electronic health records, while standardized protocols were used to collect anthropometric measurements and biological samples [13].

Baseline plasma was cryopreserved at −80°C or in liquid nitrogen. A representative subset was shipped to Olink Analysis Services (Uppsala, Sweden) for high‐throughput proteomic profiling via proximity‐extension assays. Raw signals were transformed into Normalised Protein expression (NPX) values. NPX values are log2‐normalized protein expression units provided by the UK Biobank; a 1‐unit increase in NPX approximately corresponds to a doubling of protein abundance. Of the 2923 proteins assayed, 2911 met quality‐control criteria (≥ 80% completeness). Detailed QC procedures and external validation have been published elsewhere [14].

Among 53,014 participants with Olink data, we excluded 1062 missing EDA2R values, 6304 with CVD, 226 with a cancer diagnosis, and 117 who withdrew from the UK Biobank study. The final analytic cohort comprised 45,305 individuals. This work was approved under UK Biobank application 105,322.

### 2.2 The Definition of Outcome

UK Biobank disease endpoints were ascertained through linked primary‐care records, hospital‐episode statistics, and self‐reported diagnoses, with follow‐up censored on December 31, 2022. A composite CVD event was defined by any ICD‐10 code I20–I25, I50, or I60–I64. Within this composite, we separately investigated four distinct presentations: angina (I20), acute myocardial infarction (AMI, I21), heart failure (HF, I50), and stroke (I60–I64) [15]. Mortality data were provided by the NHS Information Centre for England and Wales and the NHS Central Register for Scotland.

### 2.3 The Definition of Covariates

We selected covariates on the basis of prior publications and clinical relevance. At baseline, participants reported age, sex, ethnicity, smoking habits, and alcohol consumption. Body mass index (BMI) was calculated from measured height and weight. Resting systolic (SBP) and diastolic (DBP) blood pressures were obtained with a mercury sphygmomanometer while seated. Fasting plasma samples collected during recruitment were analysed by UK Biobank laboratories for glycated haemoglobin (HbA1c), creatinine (Cr), triglycerides (TG), total cholesterol (TC), low‐density lipoprotein cholesterol (LDL‐C), and high‐density lipoprotein cholesterol (HDL‐C). A composite socioeconomic‐status (SES) variable—low, medium, or high—was derived from self‐reported pre‐tax household income, occupation, and educational attainment [16].

### 2.4 Statistical Analysis

Variables with < 20% missingness were handled by random‐forest multiple imputation; those above this threshold were dropped. Continuous variables are summarised as mean (SD) when normally distributed and as median (IQR) otherwise; categorical variables are shown as *n* (%).

Cox proportional‐hazards models were applied to examine EDA2R in relation to incident CVD and all‐cause mortality across three levels: Model 1, unadjusted; Model 2, adjusted for age, sex, and ethnicity; Model 3, further adjusted for smoking, alcohol intake, HbA1c, SBP, creatinine, TG, TC, LDL‐C, HDL‐C, and SES. Restricted cubic splines (RCS) based on Model 3 were plotted to depict the dose–response relationships.

To model the temporal trajectory of EDA2R, we adopted a nested case–control framework [17, 18]. Incident CVD cases were matched 1:3 to CVD‐free controls by propensity scores using age, sex, ethnicity, smoking, alcohol, HbA1c, SBP, Cr, TG, TC, LDL‐C, HDL‐C, and SES. For each case, the time between baseline and diagnosis was calculated; matched controls were assigned the identical “surrogate” time. Within each stratum, covariates were regressed out and EDA2R values *z*‐standardised. LOESS smoothing was then applied to depict the trajectory of adjusted EDA2R levels across years preceding CVD onset.

A parametric survival analysis estimated years of life lost (YLL). Guided by prior work, the 10th (P10) and 90th (P90) percentiles of EDA2R were first identified. Participants were then classified into a low‐EDA2R group (≤ P10) and a high‐EDA2R group (≥ P90). Using age as the time scale and integrating the survival curves up to age 100, the cumulative survival area for each group was calculated. YLL equaled the difference between these areas. The computational approach follows the methods described in earlier studies. An age of 100 was chosen as the upper integration limit following common practice in previous studies [19, 20], as it approximates the maximum human life expectancy and enables standardized comparisons across analyses. The factors that were corrected in this analysis include those identified in Model 3 of the COX regression analysis.

Finally, mediation analysis was performed with the CMAverse package to test whether any of the remaining 2910 plasma proteins—excluding EDA2R—mediate the association between EDA2R and both incident CVD and all‐cause mortality. Proteins meeting three criteria—(i) significant mediation (*p* < 0.05/2910), (ii) directionally concordant effects with EDA2R, and (iii) the top 100 proteins with the largest mediation proportions were selected for GO enrichment analysis. GO enrichment analysis was performed using the clusterProfiler package, and *p*‐values were adjusted using the Benjamini–Hochberg method to control the false discovery rate (FDR).

All analyses were performed in R version 4.3.1 (R Foundation for Statistical Computing, Vienna, Austria). Two‐tailed *p* < 0.05 was considered statistically significant.

## 3 Result

### 3.1 Baseline Characteristics

The analytical cohort comprised 45,305 participants whose baseline characteristics are displayed in Table 1. Plasma EDA2R had a median (IQR) of −0.03 [−0.34, 0.28]. The median age was 57 years [50–63]; 55.9% were female and 44.1% male. Ethnicity was predominantly White (89.9%), with 10.1% from other backgrounds. Smoking status was never for 55.9%, former for 33.6%, and current for 10.5%. Current alcohol consumption was reported by 91.9%, never by 4.5%, and previous by 3.5%. SES was low in 39.2%, medium in 36.4%, and high in 24.5%. Median BMI was 26.6 kg/m² [24.1, 29.7]. Laboratory values (median [IQR]) were: TG 1.46 [1.03–2.12] mmol/L; TC 5.69 [4.97–6.45] mmol/L; LDL‐C 3.55 [2.99–4.15] mmol/L; HDL‐C 1.41 [1.19–1.69] mmol/L; HbA1c 35.1 [32.7–37.8] mmol/mol; SBP 138 [125–152] mmHg; Cr 70.0 [61.1–80.5] μmol/L. During follow‐up, 19.1% experienced total CVD: 2.5% AMI, 3.3% angina, 3.0% HF, 2.7% stroke, and 8.8% all‐cause death. Supporting Information S1: Table 1 compares baseline characteristics between participants in the lowest (P10) and highest (P90) EDA2R groups.

**Table 1:** Comparison of baseline characteristics of the study population.

Characteristic	Overall (*N* = 45,305)
EDA2R (median [IQR])	–0.03 [–0.34, 0.28]
Age, year (median [IQR])	57.00 [50.00, 63.00]
BMI (median [IQR])	26.61 [24.06, 29.68]
Sex (%)	
Female	25,316 (55.9)
Male	19,989 (44.1)
Ethnic (%)	
White	40,73 (89.9)
Other	4,575 (10.1)
Smoke (%)	
Never	25,303 (55.9)
Previous	15,236 (33.6)
Current	4,766 (10.5)
Drink (%)	
Never	2,061 (4.5)
Previous	1,588 (3.5)
Current	41,656 (91.9)
SES (%)	
Low SES	17,741 (39.2)
Medium SES	16,472 (36.4)
High SES	11,092 (24.5)
Biochemical (median [IQR])	
TG, mmol/L	1.46 [1.03, 2.12]
TC, mmol/L	5.69 [4.97, 6.45]
LDL, mmol/L	3.55 [2.99, 4.15]
HDL, mmol/L	1.41 [1.19, 1.69]
HbA1c, mmol/mol	35.10 [32.70, 37.80]
SBP, mmHg	138.00 [125.00, 152.00]
Creatinine, μmol/L	70.00 [61.10, 80.50]
Outcome (%)	
Total CVD = 1 (%)	8,667 (19.1)
AMI = 1 (%)	1,142 (2.5)
Angina = 1 (%)	1,477 (3.3)
HF = 1 (%)	1,349 (3.0)
Stroke = 1 (%)	1,203 (2.7)
All‐cause death = 1 (%)	3,988 (8.8)

Abbreviations: AMI, acute myocardial infarction; BMI, body mass index; CVD, cardiovascular diseases; HDL, high density lipoprotein; HbA1c, glycated hemoglobin; HF, heart failure; IQR, interquartile range; LDL, low density lipoprotein; N, Number; SES, socioeconomic status; SBP, systolic blood pressure; TG, triglyceride; TC, total cholesterol.

### 3.2 The Association of EDA2R With CVD and All‐Cause Mortality

**Figure 1:**
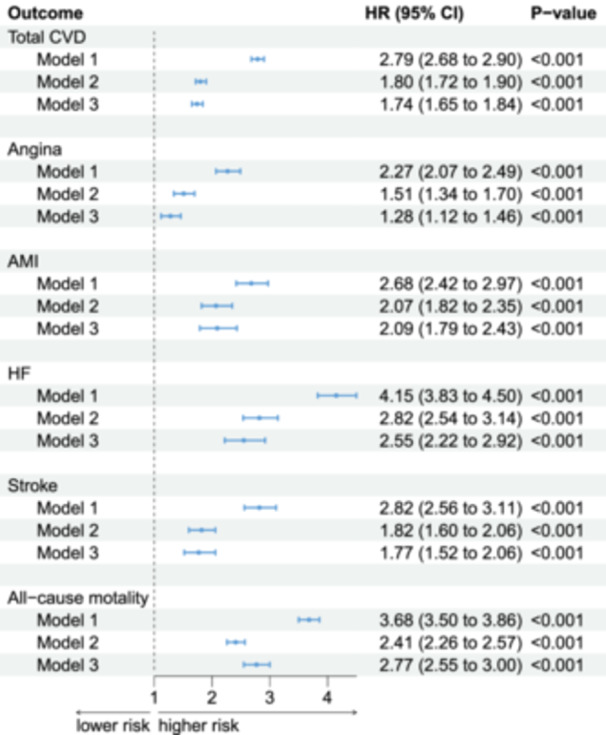
Hazard ratios for associations between plasma EDA2R levels and risk of CVD and all‐cause mortality. Three Cox proportional hazards models were used for adjustment: Model 1: Unadjusted; Model 2: Adjusted for age, sex, and ethnicity; Model 3: Adjusted for age, sex, ethnicity, BMI, smoking status, alcohol consumption, HbA1c, SBP, Cr, TG, TC, LDL, HDL, and SES.

**Figure 2:**
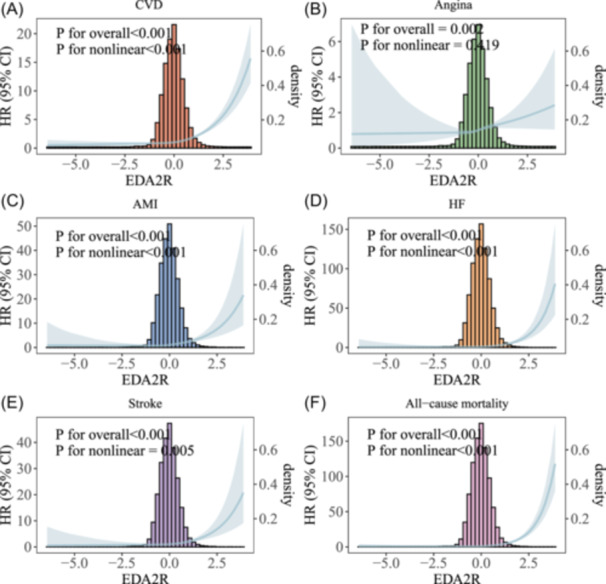
Restricted cubic spline plots of the associations between plasma EDA2R levels and risk of CVD and all‐cause mortality (fully adjusted model). (A) The relationship between EDA2R and incident total CVD. (B) The relationship between EDA2R and incident Angina. (C) The relationship between EDA2R and incident AMI. (D) The relationship between EDA2R and incident HF. (E) The relationship between EDA2R and incident stroke. (F) The relationship between EDA2R and All‐cause mortality.

Figure 1 summarises the multivariable‐adjusted associations between plasma EDA2R and six adverse outcomes. Model 3 hazard ratios (95% CI) per SD increment in EDA2R were 1.74 (1.65–1.84) for total CVD, 1.28 (1.12–1.46) for angina, 2.09 (1.79–2.43) for AMI, 2.55 (2.22–2.92) for HF, 1.77 (1.52–2.06) for stroke and 2.77 (2.55–3.00) for all‐cause mortality (all *p* < 0.001). Consistent dose–response patterns across the entire EDA2R range were corroborated by RCS in Figure 2. Higher EDA2R levels were associated with an increased risk of multiple adverse outcomes.

**Figure 3:**
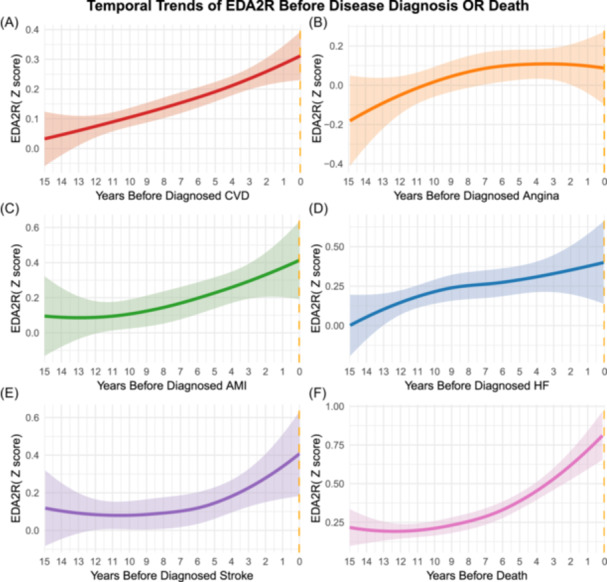
Temporal trajectories of plasma EDA2R levels (*Z*‐score) in the years preceding diagnosis of cardiovascular diseases and death. (A) Temporal trajectories of plasma EDA2R levels in the years preceding diagnosis of total CVD. (B) Temporal trajectories of plasma EDA2R levels in the years preceding diagnosis of angina. (C) Temporal trajectories of plasma EDA2R levels in the years preceding diagnosis of AMI. (D) Temporal trajectories of plasma EDA2R levels in the years preceding diagnosis of HF. (E) Temporal trajectories of plasma EDA2R Levels in the years preceding diagnosis of stroke. (F) Temporal trajectories of plasma EDA2R levels in the years preceding diagnosis of all‐cause mortality.

Figure 3 illustrates the 15‐year LOESS trajectories of plasma EDA2R preceding each clinical endpoint. Concentrations rose monotonically before total CVD, Angina, AMI, HF, stroke, and all‐cause mortality.

**Figure 4:**
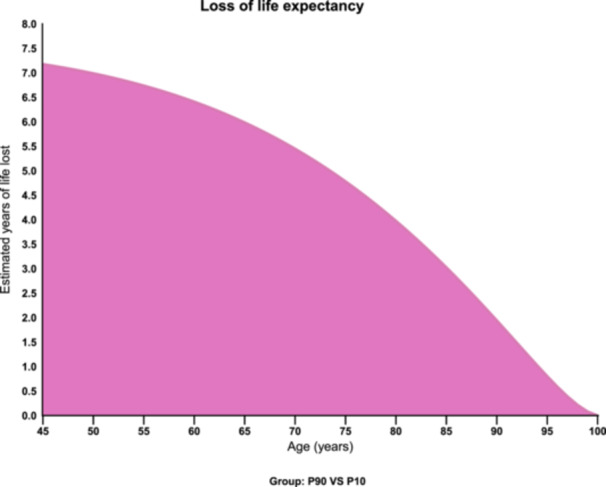
The loss of life expectancy due to the increase in EDA2R. The loss of life expectancy in the P90 group compared with the P10 group. Grouping criteria and specific values: P10 group (10th percentile): Plasma EDA2R level < ‐0.61636 (NPX). P90 group (90th percentile): Plasma EDA2R level > 0.58656 (NPX).

Participants in the highest EDA2R decile (P90) faced a pronounced reduction in remaining life expectancy relative to those in the lowest decile (P10). Parametric survival modelling estimated that this disparity amounted to 7.2 fewer life‐years at age 45, as illustrated in Figure 4.

### 3.3 Mediation Analysis and GO Enrichment Analysis

Screening the remaining 2910 plasma proteins as potential mediators revealed 302 whose mediation effects on total CVD were both statistically significant and directionally aligned with EDA2R, whereas 482 proteins met these criteria for all‐cause mortality (Supporting Information S1: Tables 2 and 3).

**Figure 5:**
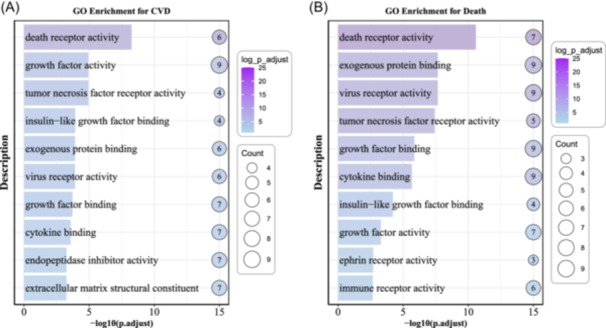
GO Enrichment analysis of mediating proteins associated with cardiovascular disease and all‐cause mortality. (A) GO terms enriched in 302 mediating proteins associated with CVD. (B) GO terms enriched in 482 mediating proteins associated with all‐cause mortality.

Figure 5 shows the GO enrichment results for the top 100 proteins that mediate the association between EDA2R and total CVD, and the top 100 proteins mediating the association between EDA2R and death.

In the CVD analysis, the enriched molecular‐function terms include death receptor activity, growth factor activity, TNF receptor activity, insulin‐like growth factor binding, exogenous protein binding, virus receptor activity, growth factor binding, cytokine binding, endopeptidase inhibitor activity, and extracellular matrix structural constituent.

In the death analysis, significantly enriched terms cover death receptor activity, exogenous protein binding, virus receptor activity, TNF receptor activity, growth factor binding, cytokine binding, growth factor activity, ephrin receptor activity, and immune receptor activity.

## 4 Discussion

In this prospective cohort study, we found that EDA2R plays a potentially important role in the occurrence and progression of CVD. Higher EDA2R expression levels were significantly positively associated with the risk of cardiovascular events such as AMI and HF. Elevated EDA2R levels were observed before the onset of disease. GO enrichment analysis indicated that pathways including “death receptor activity,” “TNF receptor activity,” and “cytokine binding” may be involved in the processes linking EDA2R to increased risk of CVD and mortality.

As a member of the tumor necrosis factor receptor superfamily (TNFRSF), EDA2R exerts important functions in multiple physiological and pathological contexts [21]. Several TNFRSF members have been confirmed to be closely associated with CVD. For example, TWEAK and its receptor Fn14 are significantly upregulated during pathological cardiovascular remodeling [22], while OPG deficiency in mice leads to aortic calcification, suggesting its protective role against medial calcification [23]. Although many TNFRSF members have been extensively investigated in CVD, research on the cardiovascular roles of EDA2R remains limited. Based on previous studies, EDA2R expression is positively correlated with cardiovascular health indicators, including troponin T, intima–media thickness, and creatinine levels, suggesting its potential as a candidate biomarker and therapeutic target for cardiometabolic aging [24]. In people living with HIV (PLHIV), EDA2R was associated with both the presence and progression risk of CVD during a 5‐year follow‐up period [11]. Furthermore, knockdown of EDA2R has been shown to inhibit activation of the NF‐κB signaling pathway and attenuate myocardial ischemia–reperfusion injury [12]. However, previous studies were generally limited by small sample sizes, often based on specific populations rather than the general population, and predominantly cross‐sectional in design, lacking large‐scale prospective evidence to validate the association between EDA2R and CVD. To address this gap, we leveraged data from a large population‐based cohort to systematically assess the relationships between EDA2R, incident CVD, and mortality, thereby providing novel evidence supporting its potential as a biomarker and therapeutic target.

GO enrichment analysis in our study revealed that EDA2R and its related genes were significantly enriched in molecular functions such as “death receptor activity,” “TNF receptor activity,” and “cytokine binding” across CVD‐ and mortality‐related phenotypes. These findings suggest that EDA2R may be involved in the onset, progression, and prognosis of cardiac diseases through mediating inflammatory responses, programmed cell death, and growth factor signaling pathways.

Of particular note, “death receptor activity” was significantly enriched in both CVD and all‐cause mortality phenotypes. Previous studies using hypoxia/reoxygenation (H/R) cell models have shown that EDA2R knockdown increases cell viability and inhibits H/R‐induced cardiomyocyte apoptosis, while also alleviating ischemia–reperfusion injury via suppression of NF‐κB signaling [12]. Similarly, Jia et al. reported that silencing EDA2R attenuated hyperoxia‐induced lung epithelial cell injury through inhibition of the NF‐κB pathway [25]. As a TNFRSF member, EDA2R can activate downstream signaling cascades such as NF‐κB and MAPK, thereby inducing caspase‐dependent apoptosis or RIPK1/RIPK3‐mediated necroptosis [12]. Thus, we speculate that EDA2R may be involved in cardiomyocyte apoptosis or necroptosis through classical death receptor pathways (such as Fas, TNFR) and NF‐κB signaling, which could contribute to cardiomyocyte loss and functional deterioration in pathological settings such as myocardial infarction or ischemia–reperfusion injury. Nonetheless, these mechanisms require further experimental validation.

In addition, the enrichment of “cytokine binding” and “immune receptor activity” suggests that EDA2R may play an important role in inflammation regulation. Previous evidence has shown that EDA2R knockdown can attenuate hyperoxia‐induced lung epithelial cell injury by suppressing NF‐κB‐mediated inflammatory responses [25]. Based on previous studies, EDA2R may be associated with enhanced responsiveness to TNF family signaling and increased secretion of pro‐inflammatory cytokines such as IL‐6 and IL‐1β [26], which could in turn amplify local chronic inflammation and contribute to inflammatory remodeling and injury responses in cardiac tissue. Furthermore, our study also observed significant enrichment of EDA2R‐related genes in “virus receptor activity” and “exogenous protein binding,” implying that EDA2R may participate in immune activation and secondary tissue injury by recognizing pathogen‐associated molecular patterns.

In summary, our findings demonstrate a strong association between EDA2R levels and the risk of both CVD and mortality. RCS and LOESS trajectory analyses further indicated that elevated EDA2R levels were positively correlated with increased risks of CVD and death, suggesting its potential utility as an early diagnostic and risk prediction biomarker for cardiovascular disease. The observed reduction in life expectancy among individuals with high EDA2R levels also supports its value as a clinical risk stratification tool. Moreover, if the observed associations are confirmed in mechanistic studies, targeting EDA2R or its downstream pathways may warrant investigation as a potential therapeutic strategy for CVD management.

### 4.1 Limitations

This study has several limitations. First, plasma EDA2R was measured only at baseline using a single blood sample, which may not fully capture long‐term exposure given potential fluctuations in protein levels over time and also limits our ability to draw causal inferences. Although the LOESS trajectory analysis provided supportive evidence of increasing EDA2R levels years before clinical events, this statistical approach cannot replace actual repeated measurements. Single‐measurement exposure may introduce non‐differential misclassification bias, which typically attenuates the observed associations toward the null. Future studies with serial proteomic measurements are warranted to validate the stability of EDA2R as a long‐term risk marker and to better characterize its temporal dynamics in relation to disease onset. In addition, the mediating proteins identified through mediation analysis are based on the assumptions of the statistical model, and the potential influence of residual confounding factors or reverse causation cannot be fully excluded. Finally, the biological functions of these proteins were inferred solely through GO enrichment analysis, without experimental validation of their specific mechanisms. Future studies should include further basic experimental investigations to confirm these findings. Fourth, the study population was predominantly of European ancestry (89.9% White), reflecting the demographic composition of the UK Biobank. This homogeneity limits the generalizability of our findings to other racial and ethnic groups, such as individuals of African, South Asian, or East Asian descent. Given that genetic background, lifestyle factors, and protein expression levels may vary across populations, future studies in more diverse cohorts are warranted to validate the external validity of the observed associations between plasma EDA2R and cardiovascular outcomes.

## 5 Conclusion

In this large‐scale prospective cohort study of 45,305 UK Biobank participants, elevated plasma EDA2R levels were consistently associated with an increased risk of incident CVD and all‐cause mortality. Mediation analysis and GO enrichment analysis indicated that hundreds of plasma proteins, mainly involved in immune and inflammatory pathways, partially mediated these associations. These findings suggest that plasma EDA2R is a promising prognostic biomarker for cardiovascular risk stratification and may warrant further investigation as a potential therapeutic target.

## Author Contributions

Yansong Guo designed the research and is the guarantor of this work, with full access to all data and responsibility for data integrity and analysis accuracy. Ziqing Ruan **c**onducted the analysis and wrote the first draft**.** Jiabin Tu, Hongli Xu, Yupeng Zhi, Chun Chen, and Kaiyang Lin revised the manuscript. All authors read and approved the final manuscript.

## Consent

All UK Biobank participants provided written informed consent, and the North West Multi‐Center Research Ethics Committee granted ethical approval (June 17, 2011 [11/NW/0382]; May 13, 2016 [16/NW/0274]). This research was performed in accordance with the principles of the Declaration of Helsinki and using the UK Biobank Resource (application No. 105322). Further details about the UK Biobank can be found on the showcase website (https://biobank.ndph.ox.ac.uk/showcase/). All authors agreed to publish the manuscript.

## Conflicts of Interest

The authors declare no conflicts of interest.

## Supporting information

Supporting File

Supporting Figures

## Data Availability

The data that support the findings of this study are available from the UK Biobank but restrictions apply to the availability of these data(ID:105,322), which were used under license for the current study, and so are not publicly available.
