# Supplementary material for: Plasma EDA2R and Risk of Cardiovascular Diseases and All‐Cause Mortality: Analysis of the UK Biobank Cohort
Source: Clin Cardiol. 2026 Apr 28;49(5):e70314. doi: 10.1002/clc.70314 (PMC13122565; doi:10.1002/clc.70314)

**Supplementary Figure 1**. Subgroup analysis of the association between EDA2R and CVD.

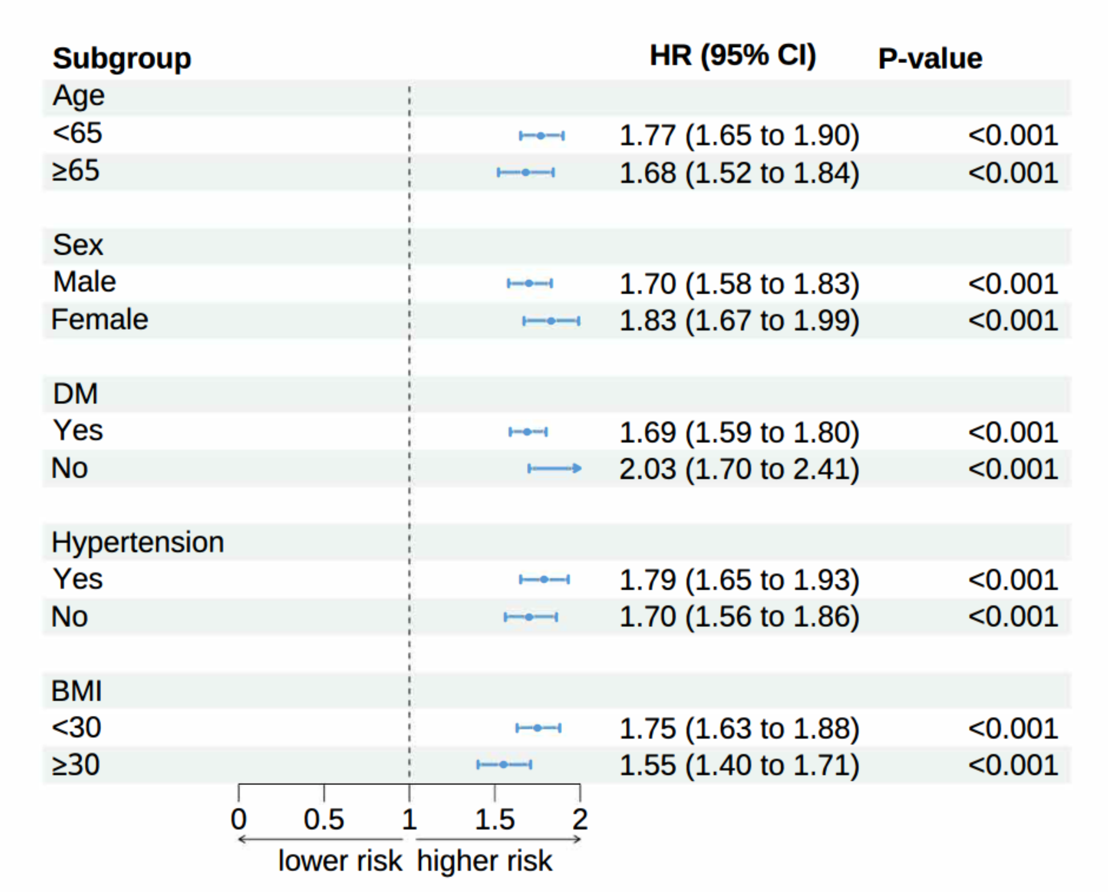

**Supplementary Figure 2**. Subgroup analysis of the association between EDA2R and All-cause mortality

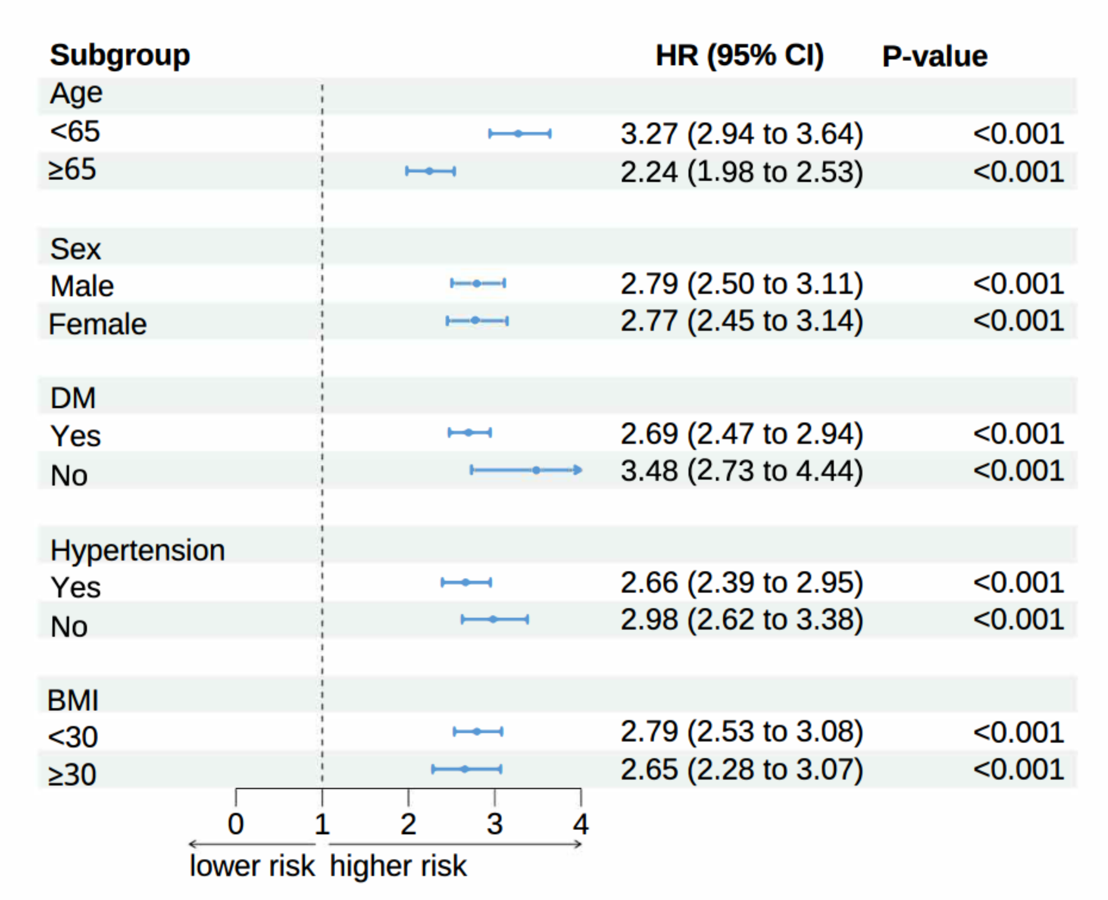

Supplement: Supplementary file 2 — Supporting Figures [file CLC-49-e70314-s001.pdf]
